# Formation of inverse Chladni patterns in liquids at microscale: roles of acoustic radiation and streaming-induced drag forces

**DOI:** 10.1007/s10404-017-1888-5

**Published:** 2017-03-03

**Authors:** Junjun Lei

**Affiliations:** Faculty of Engineering and the Environment, University of Southampton, University Road, Southampton, SO17 1BJ UK

**Keywords:** Chladni patterns, Acoustic streaming, Acoustic radiation force, Acoustofluidics, Vibrating plates

## Abstract

While Chladni patterns in air over vibrating plates at macroscale have been well studied, inverse Chladni patterns in water at microscale have recently been reported. The underlying physics for the focusing of microparticles on the vibrating interface, however, is still unclear. In this paper, we present a quantitative three-dimensional study on the acoustophoretic motion of microparticles on a clamped vibrating circular plate in contact with water with emphasis on the roles of acoustic radiation and streaming-induced drag forces. The numerical simulations show good comparisons with experimental observations and basic theory. While we provide clear demonstrations of three-dimensional particle size-dependent microparticle trajectories in vibrating plate systems, we show that acoustic radiation forces are crucial for the formation of inverse Chladni patterns in liquids on both out-of-plane and in-plane microparticle movements. For out-of-plane microparticle acoustophoresis, out-of-plane acoustic radiation forces are the main driving force in the near-field, which prevent out-of-plane acoustic streaming vortices from dragging particles away from the vibrating interface. For in-plane acoustophoresis on the vibrating interface, acoustic streaming is not the only mechanism that carries microparticles to the vibrating antinodes forming inverse Chladni patterns: In-plane acoustic radiation forces could have a greater contribution. To facilitate the design of lab-on-a-chip devices for a wide range of applications, the effects of many key parameters, including the plate radius *R* and thickness *h* and the fluid viscosity *μ*, on the microparticle acoustophoresis are discussed, which show that the threshold in-plane and out-of-plane particle sizes balanced from the acoustic radiation and streaming-induced drag forces scale linearly with *R* and $\sqrt \mu$, but inversely with $\sqrt h$.

**Electronic supplementary material:**

The online version of this article (doi:10.1007/s10404-017-1888-5) contains supplementary material, which is available to authorized users.

## Introduction

Arranging particles and cells into desired patterns for lab-on-a-chip biological applications using ultrasonic fields, i.e. acoustophoresis, by means of bulk and surface acoustic wave techniques, have attracted increasing interest in recent years (Bruus et al. 2011; Friend and Yeo 2011). When an ultrasonic standing/travelling wave is established in a micro-channel containing an aqueous suspension of particles, two main forces act on the particles: the acoustic radiation force and the streaming-induced drag force. In most bulk and surface micro-acoustofluidic manipulation devices, the latter is generally considered to be a disturbance because it places a practical lower limit on the particle size that can be manipulated by the former (Wiklund et al. 2012; Drinkwater 2016). Nevertheless, acoustic streaming flows have been applied to play an active role in the functioning of such systems. (Hammarstrom et al. 2012, 2014; Yazdi and Ardekani 2012; Antfolk et al. 2014; Devendran et al. 2014; Ohlin et al. 2015; Cheung et al. 2014; Huang et al. 2014; Patel et al. 2014; Destgeer et al. 2016; Rogers and Neild 2011; Tang and Hu 2015; Leibacher et al. 2015; Agrawal et al. 2013, 2015).

The ability to use ultrasonic fields for manipulation of particles and fluids has a long history which can date back to many eminent scientists including Chladni (1787), Faraday (1831), Kundt and Lehmann (1874), Rayleigh (1883), King (1934), Gorkov (1962). As early as 1787, the German physicist Chladni (1787) observed that randomly distributed sand particles on a vibrating metal plate could group along the nodal lines forming a wide variety of symmetrical patterns. The various patterns formed at different modes of resonance were called Chladni figures. Chladni also reported that fine particles would move in the opposite direction, to the antinodes, which was further studied by Faraday (1831), who found that it was due to air currents in the vicinity of the plate, i.e. acoustic streaming. The latter phenomenon was revisited by Van Gerner et al. (2010, 2011) who showed that it will always occur when the acceleration of the resonating plate is lower than gravity acceleration. Zhou et al. (2016) recently proposed an approach which is able to control the motion of multiple objects simultaneously and independently on a Chladni plate.

Recently, Vuillermet et al. (2016) demonstrated that it is possible to form two-dimensional inverse Chladni patterns on a vibrating circular plate in water at microscale, which extended an earlier work from Dorrestijn et al. (2007), who showed formation of one-dimensional (1D) Chladni patterns on a vibrating cantilever submerged in water, where microparticles and nanoparticles were found to move to the antinodes and nodes of the vibrating interface, respectively. Both works have depicted the two-dimensional streaming field in the near-field and emphasized the effects of in-plane streaming flow on the collections of particles at vibrating antinodes or nodes. Practical manipulation on vibrating plates, however, is three-dimensional (3D) including out-of-plane and in-plane manipulation, and interestingly, in such systems, little work has been done on the impact of acoustic radiation forces, the main engine for particle and cell manipulation in other acoustofluidic manipulation devices. Unlike microparticle acoustophoresis in bulk and surface standing wave devices that have been well studied (Barnkob et al. 2012; Muller et al. 2012, 2013; Lei et al. 2014; Hahn et al. 2015; Nama et al. 2015; Oberti et al. 2009), the literature is lacking a quantitative analysis of microparticle acoustophoresis over vibrating plate systems.

**Fig. 1:**
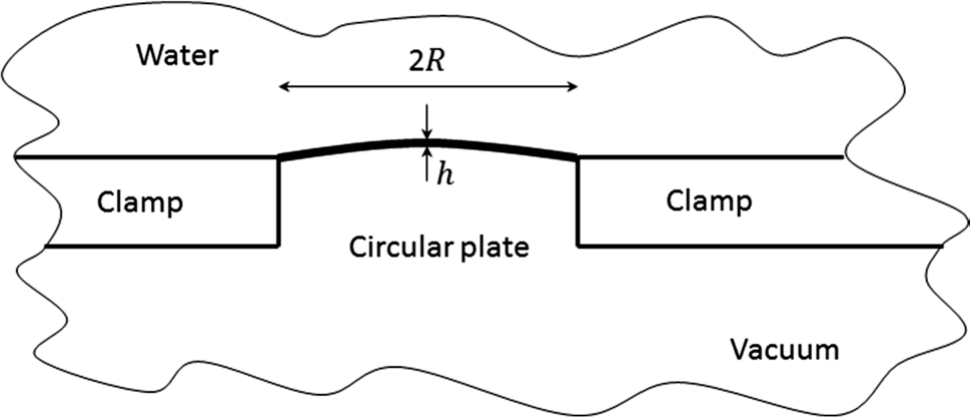
Sketch of a clamped vibrating circular plate in contact with water, where $R$ and $h$ are the radius and thickness of the circular plate, respectively

In this paper, we will show a detailed 3D study on the main forces for the formation of inverse Chladni patterns on a clamped vibrating circular plate in contact with water (see Fig. 1 for the configuration). Both out-of-plane and in-plane microparticle acoustophoresis are discussed and the contributions of main driving forces are compared, which enables a clear presentation of the underlying physics of microparticle manipulation in such systems. The many key parameters, including the plate thickness and radius, the vibration amplitude and the fluid viscosity, on the microparticle acoustophoresis are discussed. We believe that this work could provide an excellent tool on analysing microparticle acoustophoresis in vibrating plate systems and on guiding device designs for the better control of patterning of microparticles at various sizes as well as for single particle and cell manipulation.

## Numerical method

We use bold and normal-emphasis fonts to represent vector and scalar quantities, respectively. Here, we assume a homogeneous isotropic fluid, in which the continuity and momentum equations for the fluid motion are.

1a $$\frac{\partial \rho }{\partial t} + \nabla \cdot \left( {\rho \varvec{u}} \right) = 0,$$

1b $$\rho \left( {\frac{{\partial \varvec{u}}}{\partial t} + \varvec{u} \cdot \nabla \varvec{u}} \right) = - \nabla p + \mu \nabla^{2} \varvec{u} + \left( {\mu_{b} + \frac{1}{3}\mu } \right)\nabla \nabla \cdot \varvec{u},$$

where $\rho$ is the fluid density, *t* is time, $\varvec{u}$ is the fluid velocity, *p* is the pressure and *μ* and *μ*_*b*_ are, respectively, the dynamic and bulk viscosity coefficients of the fluid.

Taking the first and second order into account, we write the perturbation series of fluid density, pressure and velocity: (Bruus 2012)

2a $$\rho = \rho_{0} + \rho_{1} + \rho_{2} ,$$

2b $$p = p_{0} + p_{1} + p_{2} ,$$

2c $$\varvec{u} = \varvec{u}_{1} + \varvec{u}_{2} ,$$

where the subscripts 0, 1 and 2 represent the static (absence of sound), first-order and second-order quantities, respectively. Substituting Eq. (2) into Eq. (1) and considering the equations to the first order, Eq. (1) for solving the first-order acoustic velocity take the form,

3a $$\frac{{\partial \rho_{1} }}{\partial t} + \rho_{0} \nabla \cdot \varvec{u}_{1} = 0,$$

3b $$\rho_{0} \frac{{\partial \varvec{u}_{{\mathbf{1}}} }}{\partial t} = - \nabla p_{1} + \mu \nabla^{2} \varvec{u}_{{\mathbf{1}}} + \left( {\mu_{b} + \frac{1}{3}\mu } \right)\nabla \nabla \cdot \varvec{u}_{{\mathbf{1}}} .$$

Repeating the above procedure, considering the equations to the second order and taking the time average of Eq. (1) using Eq. (2), the continuity and momentum equations for solving the second-order time-averaged acoustic streaming velocity can be turned into

4a $$\nabla \cdot \overline{{\rho_{1} \varvec{u}_{{\mathbf{1}}} }} + \rho_{0} \nabla \cdot \overline{{\varvec{u}_{{\mathbf{2}}} }} = 0,$$

4b $$- \nabla \overline{{p_{2} }} + \mu \nabla^{2} \overline{{\varvec{u}_{{\mathbf{2}}} }} + \left( {\mu_{b} + \frac{1}{3}\mu } \right)\nabla \nabla \cdot \overline{{\varvec{u}_{{\mathbf{2}}} }} + \varvec{F} = 0,$$

4c $$\varvec{F} = - \rho_{0} \overline{{\varvec{u}_{{\mathbf{1}}} \nabla \cdot \varvec{u}_{{\mathbf{1}}} + \varvec{u}_{{\mathbf{1}}} \cdot \nabla \varvec{u}_{{\mathbf{1}}} }} ,$$

where the upper bar denotes a time-averaged value and $\varvec{F}$ is the Reynolds stress force (Lighthill 1978). When modelling the steady-state streaming flows in most practical acoustofluidic manipulation devices, the inertial force $\overline{{\varvec{u}_{{\mathbf{2}}} }} \cdot \nabla \overline{{\varvec{u}_{{\mathbf{2}}} }}$ is generally negligible compared to the viscosity force in such systems, which results in the creeping motion. The divergence-free velocity $\overline{{\varvec{u}_{{\mathbf{2}}}^{\varvec{M}} }} = \overline{{\varvec{u}_{{\mathbf{2}}} }} + \overline{{\rho_{1} \varvec{u}_{{\mathbf{1}}} }} /\rho_{0}$, derived from Eq. (4a), is the mass transport velocity of the acoustic streaming, which is generally closer to the velocity of tracer particles in a streaming flow than $\overline{{\varvec{u}_{2} }}$ (Nyborg 1998).

In this work, only the boundary-driven streaming field was solved because an evanescent wave field is established (see below) such that the overall streaming field is dominated by the boundary-driven streaming. Moreover, as the inner streaming vortices are confined only at the thin viscous boundary layer [thickness of $\delta_{v} \approx 0.6$ µm at 1 MHz in water (Bruus 2012)], for numerical efficiency, we solved only the 3D outer streaming fields using Nyborg’s limiting velocity method (Nyborg 1958; Lee and Wang 1989) as those published previously (Lei et al. 2013, 2014, 2016). Although the inner streaming fields were not computed in this work, they can, of course, be known from the limiting velocity field.

## Numerical model, results and discussion

**Fig. 2:**
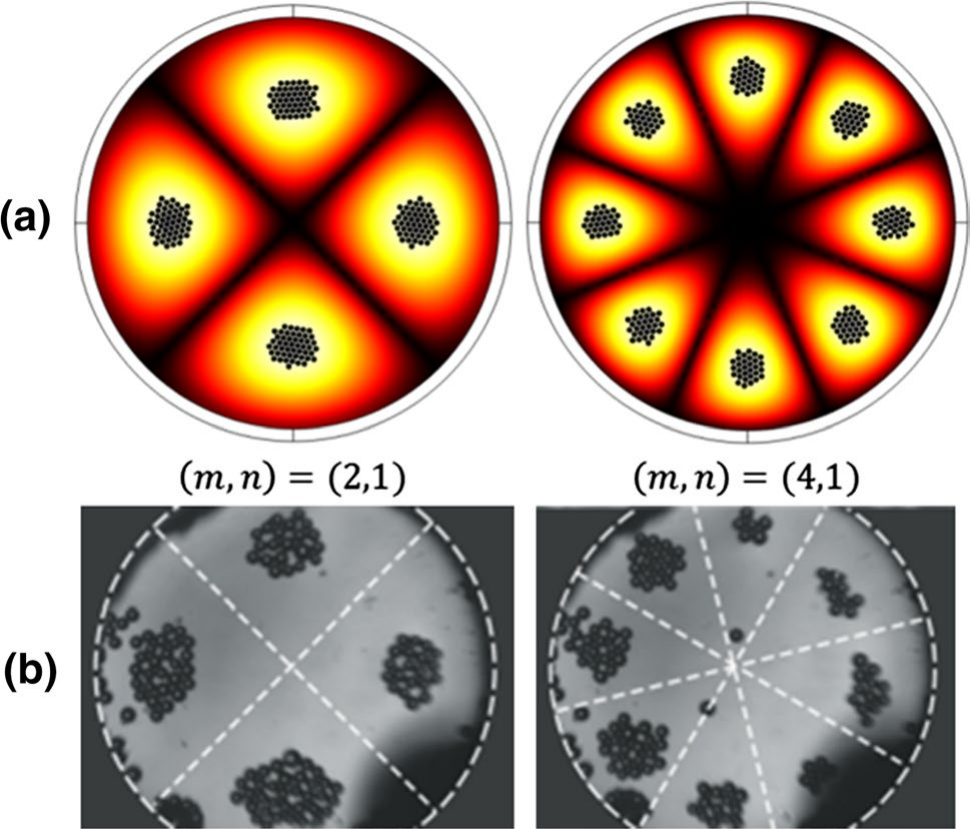
(Colour online) *Top views* of the final positions of microparticles (radius of 30 µm) on a plate at various vibrating modes: **a** modelled, where *spheres* are the microparticles and *colours* show the vibrating displacements (*white* for maximum and *black* for zero); and **b** measured, adapted with permission from Vuillermet et al. (2016) Copyrighted by the American Physical Society. The particle properties used in simulations are included in Table 1

**Fig. 3:**
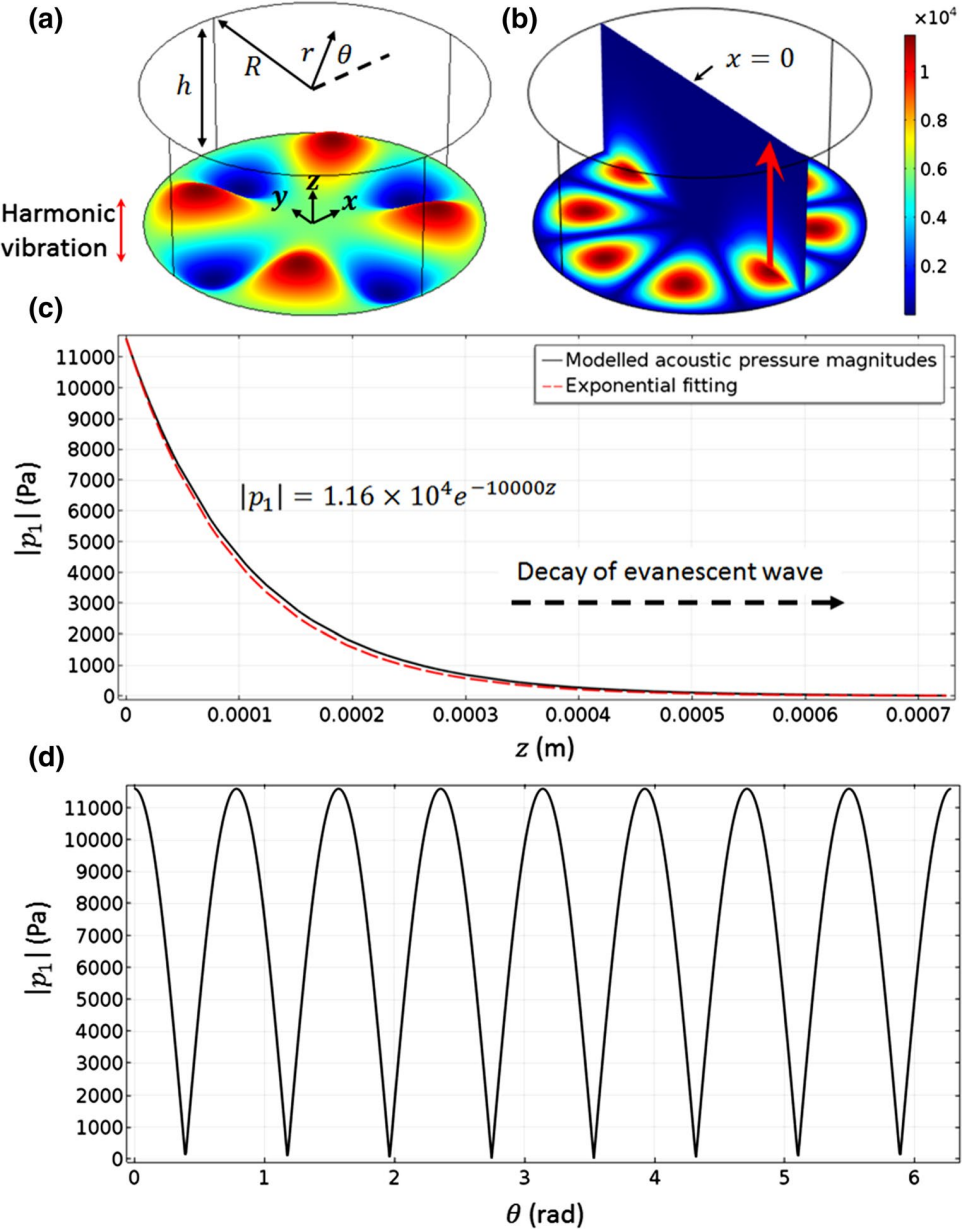
(Colour online) **a** Geometry of the considered problem, where the bottom edge ($z = 0$) vibrates at a (4, 1) mode; **b** 3D acoustic pressure magnitudes ($\left| {p_{1} } \right|$, Pa); **c** out-of-plane $\left| {p_{1} } \right|$ [*arrow* in (**b**)]; and **d** in-plane $\left| {p_{1} } \right|$ on $r = 0.56$ mm at the bottom edge. $r = \sqrt {x^{2} + y^{2} }$ and $\theta = { \arctan }\left( {y/x} \right)$. The *dashed line* and the *equation* in (**c**) show the exponential fitting of the modelled acoustic pressure magnitudes

To validate the numerical results, a clamped circular plate of radius *R* = 800 µm and thickness *h* = 5.9 µm was firstly considered, which has a same size to the one used in Vuillermet et al.’s experiments (2016). Our model is slightly different to the device in Vuillermet et al.’s experiments. It can be seen from Fig. 1 that our model shows a vibrating clamped plate in a free space while the side boundaries of Vuillermet et al.’s device have sound reflections, which may result in acoustic pressure antinodes at the plate boundaries. More model parameters are found in Table 1. The model configuration is shown in Fig. 3a, where a cylindrical fluid-channel-only model was considered. Cartesian ($x, y, z$) and cylindrical ($r, \theta , z$) coordinates were used for the convenience of calculations. The finite element package COMSOL 5.2 (COMSOL Multiphysics 2015) was used to solve all equations. The modelled final particle (radius of 30 µm) positions driven by the main forces including acoustic radiation forces, streaming-induced drag forces and buoyancy forces at two vibrating modes are shown in Fig. 2a. It can be seen that the inverse Chladni patterns the microparticles form compare well with Vuillermet et al.’s (2016) experimental observations. In the following, we will show step by step why microparticles are gathered to the vibrating antinodes forming inverse Chladni patterns and the contributions of various driving forces on the acoustophoretic motion of microparticles at various sizes.

**Table 1:** Model parameters

Parameter	Symbol	Value	Unit
Model domain	$\pi R^{2} \times h$	$\pi \times 0.8^{2} \times 0.725$	mm^3^
Density of plate	$\rho$	2320	kg m^−3^
Plate Poisson’s ratio	$\upsilon$	0.22	
Plate Young’s modulus	$E$	169	GPa
Sound speed in plate	$u$	55	m s^−1^
Particle density	$\rho_{p}$	1050	kg m^−3^
Sound speed in particle	$c_{p}$	1960	m s^−1^
Density of water	$\rho_{f}$	1000	kg m^−3^
Sound speed in water	$c_{f}$	1480	m s^−1^

It is noteworthy that we have previously applied a fluid-channel-only model to study the 3D transducer-plane streaming fields in bulk acoustofluidic manipulation devices (Lei 2015), where the excitation of transducer was approximated by a Gaussian distribution of boundary vibration. The fluid-channel-only model applied in this work has more merits because we can easily write down the displacement equation when the circular plate vibrates at a resonant mode (see Eq. (6) below), and thus, there is no need to make an approximation on the boundary vibrations as we did in the previous models (Lei et al. 2013, 2016).

### Resonant frequencies

Resonant frequencies at various modes were firstly modelled, which are shown in Table 2. For comparison, the modelled eigenfrequencies of first eight modes for another two cases, namely no load and load with air, are also presented. It can be seen that the resonant frequencies for vibrations in air and those in vacuum are very close; differences are small enough to be considered as numerical errors, suggesting that omitting the influence of air does not introduce any significant error on the resonant frequencies. The resonant frequencies of vibration in contact with water, however, have been reduced at least by a factor of 3 for all the modes presented, which means that we have to consider the influence of external load introduced by the surrounding water. All the results shown in this paper are for the (4, 1) mode ($\delta_{v} \approx 1.84$ µm) unless otherwise stated.

**Table 2:** The modelled resonant frequencies (Hz) of first eight modes for various loads

Modes	No load	Air	Water
(0, 1)	37,772	37,270	–
(1, 1)	78,447	77,562	16,320
(2, 1)	128,390	127,330	33,660
(0, 2)	146,330	145,400	35,122
(3, 1)	187,340	186,100	55,884
(1, 2)	223,070	221,900	65,745
(4, 1)	255,100	253,660	83,448
(2, 2)	309,350	307,930	102,580

The computations were performed on a Lenovo Y50 running Windows 8 (64-bit) equipped with 16 GB RAM and Intel(R) Core(TM) i7-4710HQ processor of clock frequency 2.5 GHz. The mesh constitution was chosen based on the method described in a previous work (Lei et al. 2013), which chooses the mesh size to obtain steady solutions, i.e. ensuring further refining of mesh does not change the solution significantly. This model resulted in 131,521 mesh elements, a peak RAM usage of 4.96 GB (at the acoustic step), and a running time of about 4 h for solving the steps described between Sects. 3.2 and 3.6 below.

### First-order acoustic fields

The first-order acoustic fields were modelled using the COMSOL ‘*Pressure Acoustics, Frequency Domain*’ interface, which solves the harmonic, linearized acoustic problem, taking the form,

5 $$\nabla^{2} p_{1} + \frac{{\omega^{2} }}{{c^{2} }}p_{1} = 0,$$

where *ω* is the angular frequency and *c* is the speed of sound in the fluid. The acoustic fields in the model regime were created by a harmonic vibration of the bottom edge (i.e. the plate) coupled with radiation boundary conditions on all other edges. For comparison, we also tried adding perfect matching layers around the cylindrical domain to absorb all outgoing waves and found that the differences on all the modelled quantities between these two methods are within 3%. To give a clear presentation of results, we show here the results modelled form radiation boundary conditions.

For a $\left( {m, n} \right)$ vibrating mode, the plate displacement amplitude can be written as

6 $$w = J_{m} \left( {\frac{{\alpha_{mn} }}{R}r} \right)\cos \left( {m\theta } \right),$$

where $J_{m} \left( \cdot \right)$ is the Bessel function of the first kind of order *m* and $\alpha_{mn}$ is the *n*th zero of $J_{m} \left( \cdot \right)$. The results presented in this paper were obtained at a vibration amplitude of 0.4 µm unless otherwise stated. The vibration amplitude has a limited effect on the shape of microparticle trajectories as both the acoustic radiation force and streaming-induced drag force scale with the square of the vibration amplitude (more discussions can be found below).

As shown in Fig. 3b, a standing wave field was established on the vibrating interface with acoustic pressure nodes and antinodes locating at plate displacement nodes and antinodes, respectively. The standing wave field is shown more clearly in Fig. 3d, where the in-plane circumferential acoustic pressure magnitudes are plotted. The out-of-plane acoustic pressure magnitudes over a vibrating antinode are plotted in Fig. 3c, which shows that the acoustic pressure magnitudes decay exponentially with the increase in distance from the vibrating interface. The reason is that the plate wave travels at the vibrating interface at a subsonic regime leading to an evanescent wave field: the plate wave velocity at substrate surface $u = \lambda f_{r} \approx 55$ m/s $\ll u_{l}$, where *λ* is the acoustic wavelength, *f*_*r*_ is the resonant frequency and *u*_*l*_ is the speed of sound in the liquid.

### Acoustic radiation forces

The corresponding 3D acoustic radiation forces were solved from the Gorkov equation (Gorkov 1962),

7 $$\varvec{F}_{{\varvec{ac}}} = \nabla \left\{ {V_{0} \left[ {\frac{{3\left( {\rho_{p} - \rho_{f} } \right)}}{{2\rho_{p} + \rho_{f} }}\overline{{E_{kin} }} - \left( {1 - \frac{{\beta_{p} }}{{\beta_{f} }}} \right)\overline{{E_{pot} }} } \right]} \right\},$$

where $\overline{{E_{kin} }}$ and $\overline{{E_{pot} }}$ are the time-averaged kinematic and potential energy, $\rho_{p}$ and $\rho_{f}$ are, respectively, the density of particle and fluid, $\beta_{p} = 1/\left( {\rho_{p} c_{p}^{2} } \right)$ and $\beta_{f} = 1/\left( {\rho_{f} c_{f}^{2} } \right)$ are the compressibility of particle and fluid, and $V_{0}$ is the particle volume (see Table 1 for model properties). Equation (7) is valid for particles that are small compared to the acoustic wavelength *λ* in the limit $r_{0} /\lambda \ll 1$ (where *r*_0_ is the radius of the particle) in an inviscid fluid in an arbitrary sound field. (Gorkov 1962) When a particle moves close to the vibrating plate, the acoustic radiation forces may oscillate weakly with a decrease in distance to the plate due to the multiple-scattering interaction and wall interference, while the force magnitudes will not be significantly affected (Wang and Dual 2012).

**Fig. 4:**
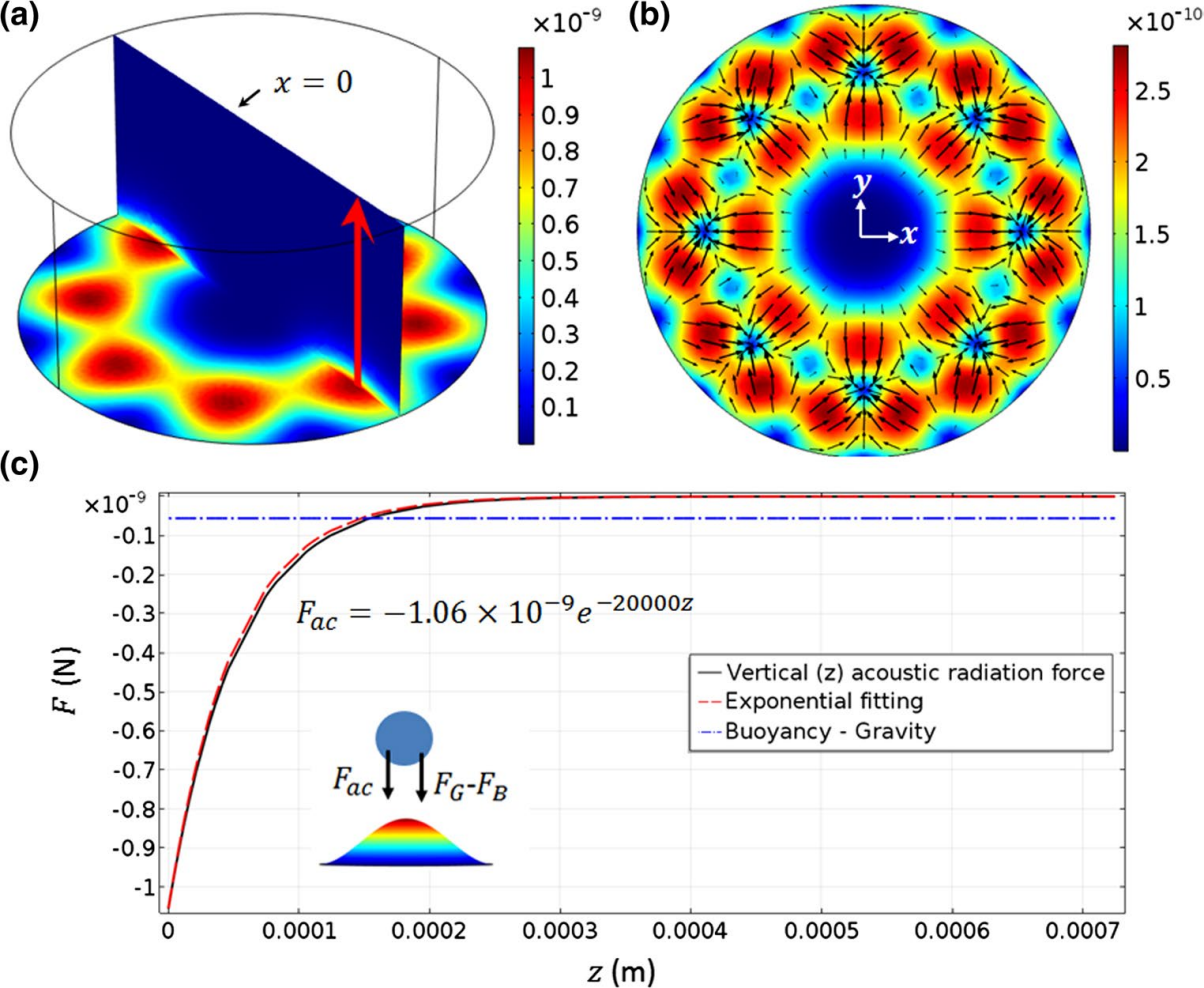
(Colour online) **a** 3D acoustic radiation force magnitudes ($\left| {F_{ac} } \right|$, N) on a particle with a radius of 30 µm; **b** in-plane $\left| {F_{ac} } \right|$; and **c** out-of-plane $\left| {F_{ac} } \right|$ [*red arrow* in (**a**)], where the *inset* shows the directions of the plotted forces above a vibrating antinode. $F_{B}$ and $F_{G}$ are the buoyancy and gravity, respectively. The *dashed line* and the *equation* in (**c**) show the exponential fitting of the modelled acoustic radiation force

The modelled acoustic radiation force fields are shown in Fig. 4. As shown in Fig. 4c, the out-of-plane acoustic radiation forces also decrease exponentially with the increase in distance from the vibrating interface. In the near-field, at this vibrating amplitude, the out-of-plane acoustic radiation forces have a greater contribution on the sedimentation of microparticles than the buoyancy forces. With an increase in vibration amplitude, we can expect dominant out-of-plane acoustic radiation forces over buoyancy forces. Interestingly, as shown in Fig. 4b, the in-plane acoustic radiation forces carry microparticles away from the acoustic pressure nodes and converge at antinodes from all directions, in contrast with the conditions usually found in bulk and surface standing wave manipulation devices, where the acoustic radiation forces move most particles and cells of interest to the acoustic pressure nodes (Glynne-Jones et al. 2012). Examining Eq. (7), it can be seen that the acoustic radiation force is a gradient of the force potential, which contains a positive contribution from the kinematic energy (weighted by a function of the fluid and particle densities) and a negative contribution from the potential energy (weighted by a function of the fluid and particle compressibility). Comparing the contributions of these two terms in this model, it was found that the kinetic energy term dominates in the force potential, which drives microparticles to the vibrating antinodes.

### Acoustic streaming fields

The 3D acoustic streaming field was modelled using Nyborg’s limiting velocity method (Nyborg 1958; Lee and Wang 1989). It was shown that if the boundary has a radius of curvature that is much larger than the acoustic boundary layer, then the time-averaged velocity at the extremity of the inner streaming (the ‘limiting velocity’) can be approximated as a function of the local, first-order linear acoustic field. The outer streaming in the bulk of the fluid can then be predicted by a fluidic model that takes the limiting velocity as a boundary condition. The applicability and viability of the limiting velocity method have been further discussed recently (Lei et al. 2017). In Cartesian coordinates, the limiting velocity field at the driving boundaries ($z = 0$) can be written as

8a $$u_{L} = - \frac{1}{4\omega }\text{Re} \left\{ {q_{x} + u_{1}^{*} \left[ {\left( {2 + i} \right)\nabla \cdot \varvec{u}_{{\mathbf{1}}} - \left( {2 + 3i} \right)\frac{{dw_{1} }}{dz}} \right]} \right\},$$

8b $$v_{L} = - \frac{1}{4\omega }\text{Re} \left\{ {q_{y} + v_{1}^{*} \left[ {\left( {2 + i} \right)\nabla \cdot \varvec{u}_{{\mathbf{1}}} - \left( {2 + 3i} \right)\frac{{dw_{1} }}{dz}} \right]} \right\},$$

8c $$q_{x} = u_{1} \frac{{du_{1}^{*} }}{dx} + v_{1} \frac{{du_{1}^{*} }}{dy},$$

8d $$q_{y} = u_{1} \frac{{dv_{1}^{*} }}{dx} + v_{1} \frac{{dv_{1}^{*} }}{dy},$$

where *u*_*L*_ and *v*_*L*_ are the *x*- and *y*-components of the limiting velocity field, *u*_1_, *v*_1_ and *w*_1_ are the *x*-, *y*- and *z*-components of the acoustic velocity vector $\varvec{u}_{{\mathbf{1}}}$, $\text{Re} \left\{ \cdot \right\}$ denotes the real part of a complex value and $*$ is the complex conjugate.

A COMSOL ‘*Creeping Flow*’ interface was used to model the acoustic streaming field, which solves

9a $$\nabla \cdot \overline{{\varvec{u}_{{\mathbf{2}}} }} = 0,$$

9b $$\nabla p_{2} = \mu \nabla^{2} \overline{{\varvec{u}_{{\mathbf{2}}} }} .$$

**Fig. 5:**
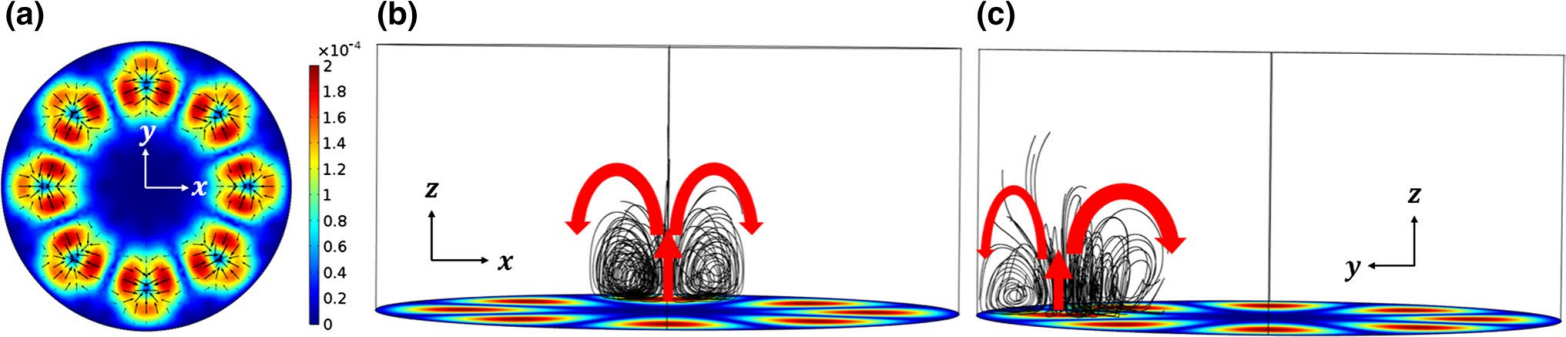
(Colour online) **a** The limiting velocity field (m/s) on the *bottom edge* ($z = 0$); and **b, c** front and left views of the 3D acoustic streaming fields, where the *colours* at the *bottom edge* in (**b, c**) show the acoustic pressure magnitudes (*red* for maximum and *blue* for zero). To give a clear presentation of the 3D acoustic streaming flows, only those above one acoustic pressure antinode are shown. *Arrows* in (**b, c**) show the streaming directions

As only outer streaming fields are solved in this method, with the assumption of low velocity and incompressible flow, the first term in the left-hand side of Eq. (4a) is zero and thus $\overline{{\varvec{u}_{{\mathbf{2}}} }} = \overline{{\varvec{u}_{{\mathbf{2}}}^{\varvec{M}} }}$ (Hamilton et al. 2003). Then, as discussed by Lighthill (1978), the Reynolds stress in the bulk of the fluid can set up hydrostatic stresses, but in the absence of attenuation these will not create vortices, hence these terms are not included in Eq. (9b). The 3D outer acoustic streaming fields in the considered model regime were generated by the limiting velocity field on the vibrating interface (see Fig. 5a) along with no-slip boundary conditions ($\overline{{\varvec{u}_{{\mathbf{2}}} }} = 0$) on all other edges.

The limiting velocity field and the 3D acoustic streaming fields are shown in Fig. 5. It can be seen that, similar to the distribution of in-plane acoustic radiation forces, the limiting velocities (i.e. the in-plane acoustic streaming velocity field) converge at the acoustic pressure antinodes from all directions leading to acoustic streaming vortices on out-of-planes perpendicular to the vibrating interface as those plotted in Fig. 5b, c, where, in order to give a clear demonstration of the 3D acoustic streaming fields, only the acoustic streaming vortices above one acoustic pressure antinode are plotted.

### Acoustic streaming-induced drag forces

Based on the acoustic streaming velocity field, we can calculate the acoustic streaming-induced drag forces on microparticles from the stokes drag,

10 $$\varvec{F}_{\varvec{d}} = 6\mu \pi r_{0} \left( {\overline{{\varvec{u}_{{\mathbf{2}}} }} - \varvec{v}} \right),$$

where $\varvec{v}$ is the particle velocity. Equation (10) is valid for particles sufficiently far from the channel walls (Happel 1965). Since microparticle acoustophoresis discussed in this work is closely associated with the vibrating plate, it is necessary to take into account the wall effect on the streaming-induced drag forces when a particle moves close to the bottom wall. When a sphere particle moves perpendicularly towards or in parallel to the vibrating plate, the streaming-induced drag force should be corrected by multiplying a wall-effect-correction factor *χ* or *γ*, respectively, which can be expressed as (Happel 1965)

11a $$\chi = \frac{4}{3}\sinh \alpha \mathop \sum \limits_{i = 1}^{\infty } \frac{{i\left( {i + 1} \right)}}{{\left( {2i - 1} \right)\left( {2i + 3} \right)}} \times \left[ {\frac{{2\sinh \left( {2i + 1} \right)\alpha + \left( {2i + 1} \right)\sinh 2\alpha }}{{4\sinh^{2} \left( {i + 1/2} \right)\alpha - \left( {2i + 1} \right)^{2} \sinh^{2} \alpha }} - 1} \right],$$

11b $$\gamma = \frac{1}{{1 - A\left( {r_{0} /H} \right) + B\left( {r_{0} /H} \right)^{3} - C\left( {r_{0} /H} \right)^{4} - D\left( {r_{0} /H} \right)^{5} }},$$

11c $$\alpha = \cosh^{ - 1} \left( {H/r_{0} } \right),$$

where $H$ is the distance from the centre of the particle to the plate and *A* = 9/16, *B* = 1/8, *C* = 45/256 and *D* = 1/16.

**Fig. 6:**
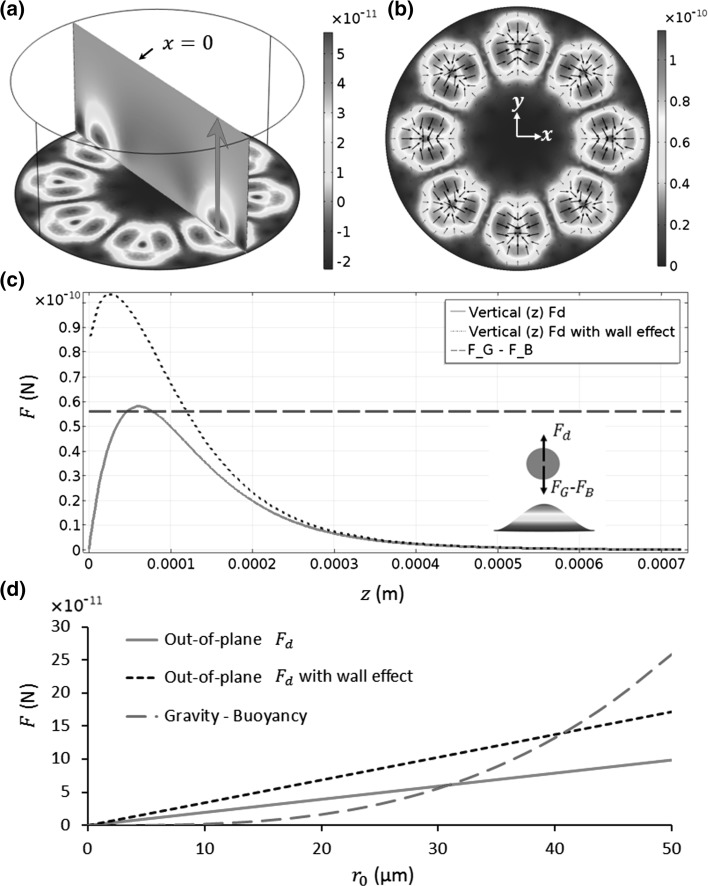
(Colour online) **a** 3D streaming-induced drag forces on a particle with a radius of 30 µm ($\left| {F_{d} } \right|$, N); **b** in-plane $\left| {F_{d} } \right|$; **c** out-of-plane $\left| {F_{d} } \right|$ [*red arrow* in (**a**)]; and **d** comparisons of maximum out-of-plane $\left| {F_{d} } \right|$ [peak in (**c**)] with the buoyancy forces for various particle sizes (radius of $r_{0}$). The *inset* in (**c**) shows the directions of the plotted forces above a vibrating antinode. $F_{B}$ and $F_{G}$ are the buoyancy and gravity, respectively

The 3D acoustic streaming-induced drag forces are shown in Fig. 6, where, for comparison, the buoyancy forces are also plotted. As shown in Fig. 6c, with the increase in distance from the vibrating interface, the out-of-plane streaming-induced drag forces rise rapidly to the maximum value in the near-field and then fall gradually to zero in the far-field. The wall effect can increase the maximum our-of-plane streaming-induced drag force by approximately a factor of 2 in this model. Also, it can be seen that, for a small vibration amplitude of *w* = 0.4 µm, the maximum out-of-plane streaming-induced drag force is larger than the buoyancy force on a particle with a radius of 30 µm. With an increase in vibration amplitude, we can expect even larger acoustic streaming-induced drag forces while the buoyancy forces remain the same. Therefore, it might be reasonable to say that introducing only the streaming effects is not enough to explain the sedimentation of microparticles, especially for those with *r*_0_ < 30 µm, where the differences between the out-of-plane streaming-induced drag forces and the buoyancy forces are even larger, as plotted in Fig. 6d, because the former and the latter scale with the particle radius and particle volume, respectively.

### Microparticle trajectories

From the acoustic radiation forces and streaming-induced drag forces that have been calculated, together with the buoyancy forces, microparticle (polystyrene beads) trajectories were modelled, following

12a $$\frac{d}{dt}\left( {m_{p} \varvec{v}} \right) = \varvec{F}_{\varvec{d}} + \varvec{F}_{{\varvec{ac}}} + \varvec{F}_{\varvec{B}} + \varvec{F}_{\varvec{G}} ,$$

12b $$\varvec{F}_{\varvec{B}} + \varvec{F}_{\varvec{G}} = \frac{4}{3}\pi r_{0}^{3} g\left( {\rho_{f} - \rho_{p} } \right),$$

where *m*_*p*_ is the particle mass, $\varvec{F}_{\varvec{B}}$ is the buoyancy, $\varvec{F}_{\varvec{G}}$ is the gravity and *g* is the gravity acceleration. In this work, it is assumed that all the forces, including acoustic radiation, streaming-induced drag and buoyancy forces, act on the centre of spherical particles (otherwise, integration of forces over the particle surface would be needed when the particles are close to the boundaries). It is noteworthy that, in addition to these main driving forces, a particle–particle interaction force was used in this model. The particle–particle interaction force can be expressed as

13 $$\varvec{F} = - k_{s} \mathop \sum \limits_{i = 1}^{N} \left( {\left| {\varvec{r} - \varvec{r}_{\varvec{i}} } \right| - r_{e} } \right)\frac{{\varvec{r} - \varvec{r}_{\varvec{i}} }}{{\varvec{r} - \varvec{r}_{\varvec{i}} }},$$

where *k*_*s*_ is the spring constant, $\varvec{r}_{\varvec{i}}$ is the position vector of the *i*th particle, and *r*_*e*_ is the equilibrium position between particles. In this model, *k*_*s*_ = 2.5 × 10^−4^ N/m for polystyrene beads (Jensenius and Zocchi 1997) and *r*_*e*_ was set as 2*r*_0_ to avoid all particles being concentrated to a single point.

**Fig. 7:**
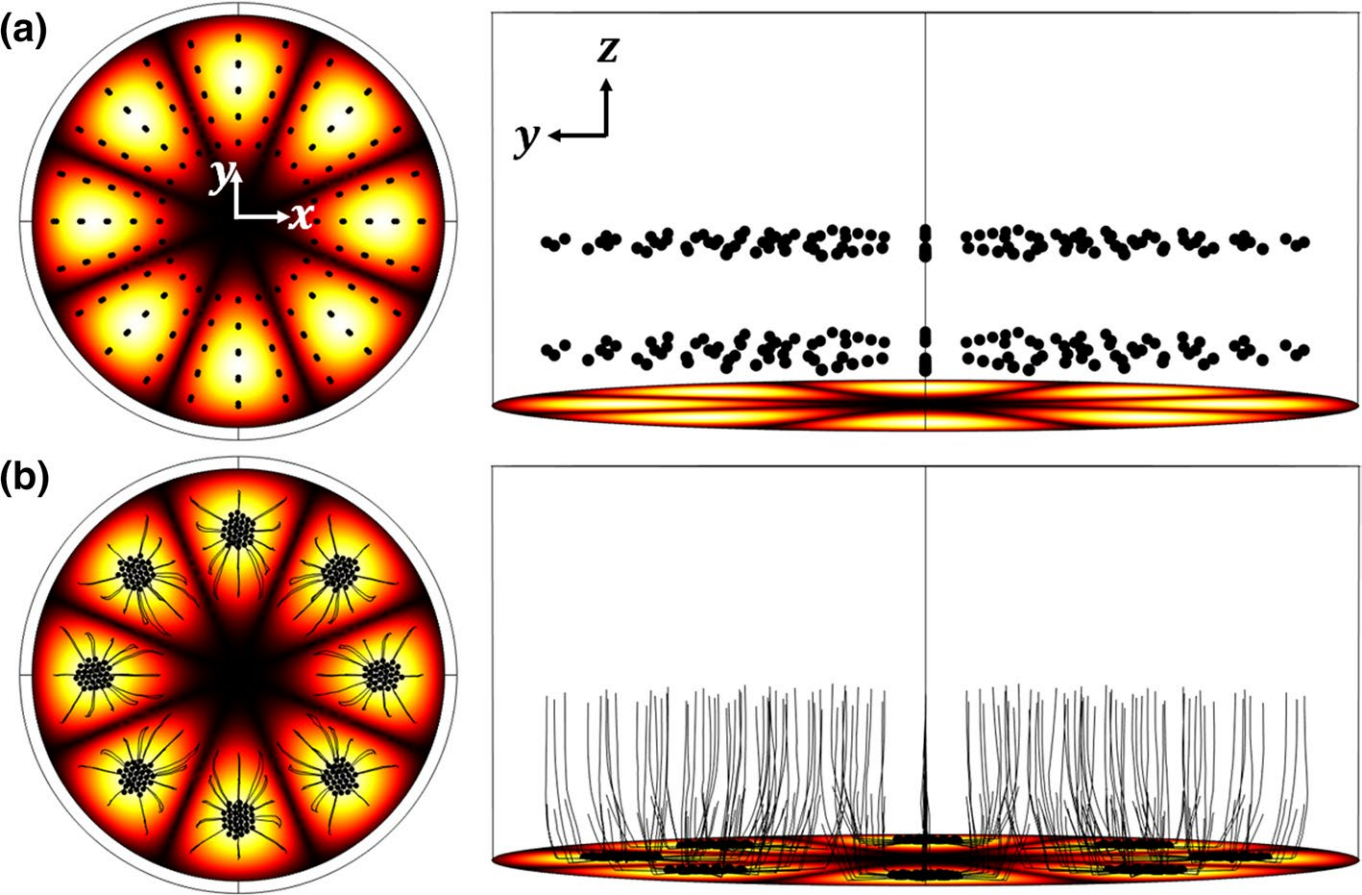
(Colour online) Trajectories of microparticles (radius of 30 µm) at: **a** $t = 0$; and **b** $t = 3$ s. *Spheres* are the microparticle, *black solid lines* show particle trajectories and *colours* at the *bottom edge* show the vibrating displacements (*white* for maximum and *black* for zero). See video in the Supplemental material

Here, a COMSOL ‘*Particle Tracing for Fluid Flow*’ interface was used to solve Eq. (12) to model the particle trajectories. The shape of the trajectories is independent of the pressure amplitude since both the acoustic radiation forces and steaming-induced drag forces scale with the square of pressure; results are presented here for an excitation amplitude of *w* = 0.4 µm. An array of tracer particles (given the properties of polystyrene beads of radius 30 µm) are seeded at $t = 0$. Acoustic radiation forces, streaming-induced drag forces and buoyancy forces act on the particles, resulting in the motion shown in Fig. 7. It can be seen that, in the considered model regime, particles with a radius of 30 µm first move towards the vibrating interface driven by the predominant out-of-plane forces and are then carried to their closest acoustic pressure antinodes by in-plane forces, resulting in *spider*-*like* trajectories and inverse Chladni patterns on the vibrating interface within seconds. Generally, particles closer to the vibrating interface take less time to settle for stronger driving forces. Particles with smaller sizes take longer to locate at the acoustic pressure antinodes for smaller driving forces and will follow the out-of-plane streaming vortices leading to acoustic streaming-dominated trajectories close to those shown in Fig. 5b, c while *r*_0_ < 6.9 µm (see explanations below and videos in the Supplemental material).

*Out-of-plane acoustophoresis.* A single particle out-of-plane acoustophoresis is directly acted upon by the acoustic radiation force, the buoyancy force and the acoustic streaming-induced drag force. The equation of motion for a spherical particle of out-of-plane velocity $\varvec{v}^{{\varvec{out}}}$ above an acoustic pressure antinode is then

14 $$\varvec{v}^{{\varvec{out}}} = \frac{{\varvec{F}_{\varvec{d}}^{{\varvec{out}}} + \varvec{F}_{{\varvec{ac}}}^{{\varvec{out}}} + \varvec{F}_{\varvec{B}} + \varvec{F}_{\varvec{G}} }}{{6\pi \mu r_{0} }}.$$

**Fig. 8:**
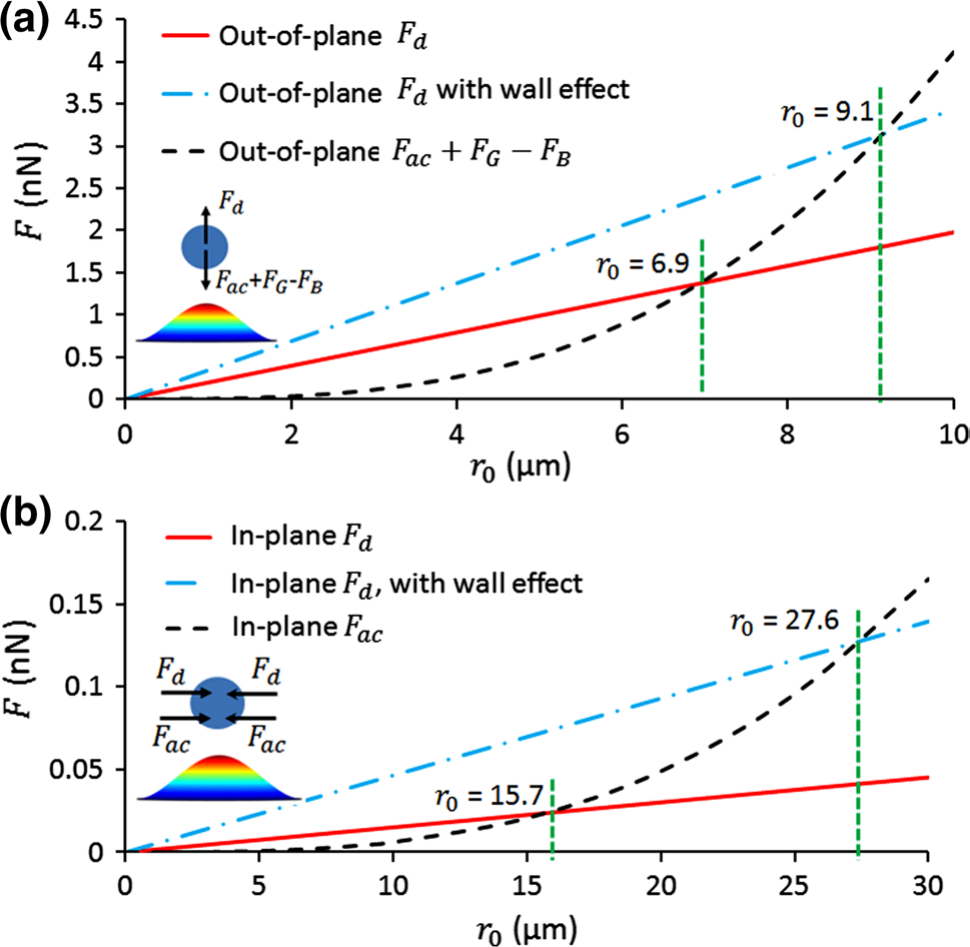
(Colour online) Comparisons of magnitudes of **a** out-of-plane forces and **b** in-plane forces on particles with various sizes (radius of $r_{0}$). The *insets* show the directions of the plotted forces above a vibrating antinode. $F_{ac}$, $F_{d}$, $F_{B}$ and $F_{G}$ are the acoustic radiation force, streaming-induced drag force, buoyancy and gravity, respectively. The in-plane forces are the average values over the bottom edge

As we have seen above, particles are concentrated at the acoustic pressure antinodes, so we take here a particle staying above an acoustic pressure antinode to analyse the contributions of the many forces on the microparticle out-of-plane acoustophoresis. As shown in the inset of Fig. 8a, the streaming-induced drag forces, $\varvec{F}_{\varvec{d}}^{{\varvec{out}}}$, competes with other forces above an acoustic pressure antinode as the acoustic streaming flow drives particles away from the pressure antinode, while other forces bring particles to the pressure antinode. Based on the fact that

15 $$\varvec{F}_{\varvec{d}}^{{\varvec{out}}} \propto r_{0}\quad {\text{and }}\quad \varvec{F}_{{\varvec{ac}}}^{{\varvec{out}}} + \varvec{F}_{\varvec{B}} + \varvec{F}_{\varvec{G}} \propto r_{0}^{3} ,$$

there should be a threshold out-of-plane particle size, $r_{0}^{out}$: for $r_{0} > r_{0}^{out}$, particles can be easily concentred to the acoustic pressure antinodes; while for $r_{0} < r_{0}^{out}$, particles will follow the out-of-plane acoustic streaming vortices. We define the threshold particle radius $r_{0}^{out}$ for crossover from these out-of-plane forces. The out-of-plane forces on particles at various sizes are plotted in Fig. 8a, which shows that, at a small vibration amplitude of *w* = 0.4 µm, the threshold particle size $r_{0}^{out} \approx 6.9$ µm. Considering the wall-effect-correction for the streaming-induced drag forces, $r_{0}^{out} \approx 9.1$ µm. This threshold out-of-plane particle size may slightly vary with the vibration amplitude as $\varvec{F}_{\varvec{B}} + \varvec{F}_{\varvec{G}}$ are independent of the vibration amplitude, while $\varvec{F}_{\varvec{d}}^{{\varvec{out}}}$ and $\varvec{F}_{{\varvec{ac}}}^{{\varvec{out}}}$ scale with the square of the vibration amplitude. As shown in Fig. 4c, the buoyancy force is approximately 1/20 of $\varvec{F}_{{\varvec{ac}}}^{{\varvec{out}}}$ at $w = 0.4$ µm on the vibrating interface. With an increase in vibration amplitude, the contribution of buoyancy force will be even smaller on the microparticle acoustophoresis in the near-field. To calculate the limit value of $r_{0}^{out}$, we can set

16 $$\varvec{F}_{{\varvec{ac}}}^{{\varvec{out}}} + \varvec{F}_{\varvec{d}}^{{\varvec{out}}} = 0$$

by ignoring the buoyancy forces, which gives

17 $$r_{0}^{out} = \sqrt {\frac{{\left| {F_{d}^{out} } \right|}}{{\left| {F_{ac}^{out} } \right|}}} r_{0} \approx 7.1 \mu m.$$

Considering the wall-effect-correction for the streaming-induced drag forces, the limit value of $r_{0}^{out} \approx 9.4$ µm.

*In-plane microparticle acoustophoresis.* For the in-plane microparticle acoustophoresis, it is acted upon by the acoustic radiation force and the streaming-induced drag force. Similar to the analyses above, the equation of motion for a spherical particle of in-plane velocity $\varvec{v}^{{\varvec{in}}}$ is then

18 $$\varvec{v}^{{\varvec{in}}} = \frac{{\varvec{F}_{\varvec{d}}^{{\varvec{in}}} + \varvec{F}_{{\varvec{ac}}}^{{\varvec{in}}} }}{{6\pi \mu r_{0} }}.$$

As shown in Figs. 4b and 6b, both the in-plane acoustic radiation force, $\varvec{F}_{{\varvec{ac}}}^{{\varvec{in}}}$, and the streaming-induced drag force, $\varvec{F}_{\varvec{d}}^{{\varvec{in}}}$, move microparticles to the acoustic pressure antinodes (see also the inset in Fig. 8b). To evaluate the contributions of these two forces on the in-plane microparticle acoustophoresis, we compare their average values over the plate interface because considering the maximum force only may not be accurate. Since both of these in-plane forces point to the acoustic pressure antinodes, they jointly contribute to the focusing of microparticles to the acoustic pressure antinodes provided that the particle sizes are big enough to avoid being driven away from the vibrating interface by out-of-plane acoustic streaming vortices (as discussed in the previous step), which could provide evidence for the much larger particle velocities measured in experiments when compared with the predicted streaming velocities as shown in Vuillermet et al. (2016) work.

Although there is no threshold in-plane particle size for the reason that both the in-plane acoustic radiation force and streaming-induced drag force drive microparticles to the acoustic pressure antinodes, we can figure out the contribution of each force on the in-plane microparticle acoustophoresis. Again, based on the fact that

19 $$\varvec{F}_{\varvec{d}}^{{\varvec{in}}} \propto r_{0}\quad {\text{and}}\quad \varvec{F}_{{\varvec{ac}}}^{{\varvec{in}}} \propto r_{0}^{3} ,$$

we can expect a critical in-plane particle size, $r_{0}^{in}$: for $r_{0} > r_{0}^{in}$, $\varvec{F}_{{\varvec{ac}}}^{{\varvec{in}}}$ contribute more to the in-plane acoustophoresis; while for $r_{0} < r_{0}^{out}$, $\varvec{F}_{\varvec{d}}^{{\varvec{in}}}$ have a higher contribution. The value of $r_{0}^{in}$ can be found from setting $\varvec{F}_{{\varvec{ac}}}^{{\varvec{in}}} = \varvec{F}_{\varvec{d}}^{{\varvec{in}}}$, which gives

20 $$r_{0}^{in} = \sqrt {\frac{{\left| {\varvec{F}_{\varvec{d}}^{{\varvec{in}}} } \right|}}{{\left| {\varvec{F}_{{\varvec{ac}}}^{{\varvec{in}}} } \right|}}} r_{0} \approx 15.7\,\upmu{\text{m}}.$$

Considering the wall-effect-correction for the streaming-induced drag forces, $r_{0}^{in} \approx 27.6$ µm. The in-plane forces on particles at various sizes are plotted in Fig. 8b. It is noteworthy that, different to the situation for $r_{0}^{out}$, $r_{0}^{in}$ is independent of the vibration amplitude $w$ because both $\varvec{F}_{{\varvec{ac}}}^{{\varvec{in}}}$ and $\varvec{F}_{\varvec{d}}^{{\varvec{in}}}$ scale with the square of $w$.

Actually, it can be seen from Eqs. (17) and (20) that, ignoring the small effect of buoyancy forces in the near-field, the relationships between the in-plane and out-of-plane threshold particle sizes and the ratios of the corresponding streaming-induced drag force and acoustic radiation force are

21 $$r_{0}^{in} , r_{0}^{out} = \sqrt {\frac{{\left| {\varvec{F}_{\varvec{d}} } \right|}}{{\left| {\varvec{F}_{{\varvec{ac}}} } \right|}}} r_{0} .$$

## Effects of key parameters on microparticle acoustophoresis

Having demonstrated the acoustophoresis of microparticles at various sizes for a particular plate (thickness of 5.9 µm and radius of 0.8 mm), in this section, we investigate the effects of many key parameters, including the plate radius and thickness and the fluid viscosity, on the performance of microparticle acoustophoresis in order to facilitate device design for a wide range of applications.

*Effects of fluid viscosity.* On the one hand, it can be seen from Eq. (8) that the magnitudes of limiting velocities (i.e. the strength of the outer streaming velocities) are independent of the fluid viscosity even though viscosity is the initial cause of acoustic streaming flows. Thus, with a change in fluid viscosity, the streaming-induced drag force, $\varvec{F}_{\varvec{d}}$, scales linearly with $\mu$, while $\varvec{F}_{{\varvec{ac}}}$ will remain the same. From Eq. (21), the following relationships are established,

22 $$r_{0}^{in} , r_{0}^{out} \propto \sqrt \mu .$$

Therefore, to eliminate the ‘side effect’ of streaming flows on the microparticle manipulation, and we can conclude that lowering the fluid viscosity is a viable way to augment the weight of acoustic radiation force on microparticle acoustophoresis.

**Fig. 9:**
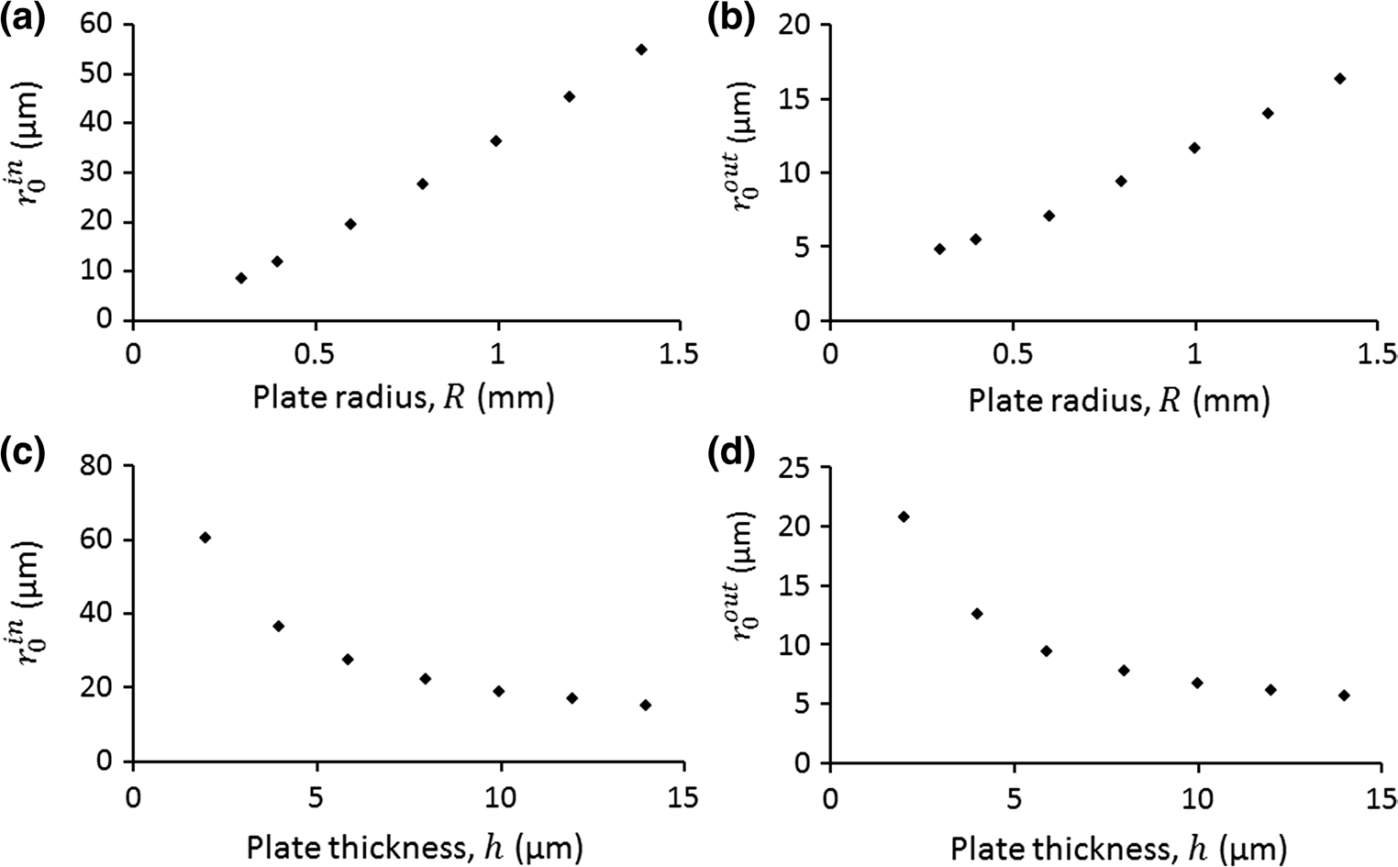
Effects of plate radius on the threshold **a** in-plane particle sizes, $r_{0}^{in}$, and **b** out-of-plane particle sizes, $r_{0}^{out}$ (with wall effect). For (**a**, **b**), the plate thickness is the same, $h = 5.9$ µm. For (**c**, **d**), the plate radius is the same, $R = 0.8$ mm

*Effects of plate thickness and radius.* To investigate the effects of plate thickness ($h$) and radius ($R$) on the microparticle acoustophoresis, we considered a series of *h* and *R* ranging from 2 to 14 µm and 0.3 to 1.4 mm, respectively. When one parameter was studied, the other parameter was kept the same. For each case, following the whole numerical procedure described in the sections above, we calculated the threshold in-plane and out-of-plane particle sizes, which are shown in Fig. 9. It can be seen that these two threshold particle sizes have similar variation tendencies: they grow with the increase in *R* and fall with the rise of *h*.

*Compare with basic theory.* Turning to the theoretical aspect, as seen from Eq. (21), to determine how these two threshold particle sizes change with the many key parameters, we only need to figure out how the force ratio on the right-hand side varies with these parameters. If we define $\varvec{v}^{{\varvec{rad}}}$ as the contribution of acoustic radiation force on the particle velocity, considering Eqs. (15) and (19), we have

23 $$\frac{{\left| {\varvec{F}_{\varvec{d}} } \right|}}{{\left| {\varvec{F}_{{\varvec{ac}}} } \right|}} = \frac{{\left| {\overline{{\varvec{u}_{{\mathbf{2}}} }} } \right|}}{{\left| {\varvec{v}^{{\varvec{rad}}} } \right|}}.$$

Examining the acoustic field in the near-field, it can be seen from Fig. 3b that, if expanded in the radial direction, the acoustic pressure field (as plotted in Fig. 3d) can be approximated to a 1D standing wave on all circumferences for $0 < r \ll R$, in which the right-hand side of Eq. (23) has the following relation (Barnkob et al. 2012)

24 $$\frac{{\left| {\overline{{\varvec{u}_{{\mathbf{2}}} }} } \right|}}{{\left| {\varvec{v}^{{\varvec{rad}}} } \right|}} = \frac{6\mu }{{\varPhi \rho_{f} \omega r_{0}^{2} }},$$

where $\varPhi \approx 0.1685$ in this work is the acoustic contrast factor and the thermoviscous effects are not included.

For a clamped circular plate with radius of $R$ and thickness of *h*, the angular frequency for an unloaded case for each $\left( {m, n} \right)$ mode follows (Leissa 1993)

25 $$\omega = \frac{{\alpha_{mn}^{2} }}{{R^{2} }}\sqrt {\frac{{Eh^{2} }}{{12\rho \left( {1 - \upsilon^{2} } \right)}}} ,$$

where $E$ is the plate Young’s modulus, *ρ* is the plate density and *υ* is the plate Poisson’s ratio. Considering the surrounding water, for a given $\left( {m, n} \right)$ mode, the angular frequency is reduced to

26a $$\omega = \frac{{\alpha_{mn}^{2} }}{{R^{2} }}\sqrt {\frac{{Eh^{2} }}{{12\rho \left( {1 - \upsilon^{2} } \right)}}} \frac{1}{C},$$

26b $$C = \sqrt {1 + \varGamma_{mn} \frac{{\rho_{f} }}{\rho }\frac{R}{h}} ,$$

where $\varGamma_{mn}$ is the non-dimensional added virtual mass incremental (NAVMI) factor, values of which can be found in Ref. (Amabili and Kwak 1996), Table 5, in the case of a clamped plate.

Combining Eqs. (21), (23), (24) and (26), the relationships between the threshold in-plane particle sizes and the many key parameters in a 1D standing wave field can be expressed as

27 $$r_{0}^{in} = Rh^{ - 0.5} \left( {\frac{6\mu C}{{\varPhi \rho_{f} \alpha_{mn}^{2} }}} \right)^{0.5} \left[ {\frac{E}{{12\rho \left( {1 - \upsilon^{2} } \right)}}} \right]^{ - 0.25} .$$

**Fig. 10:**
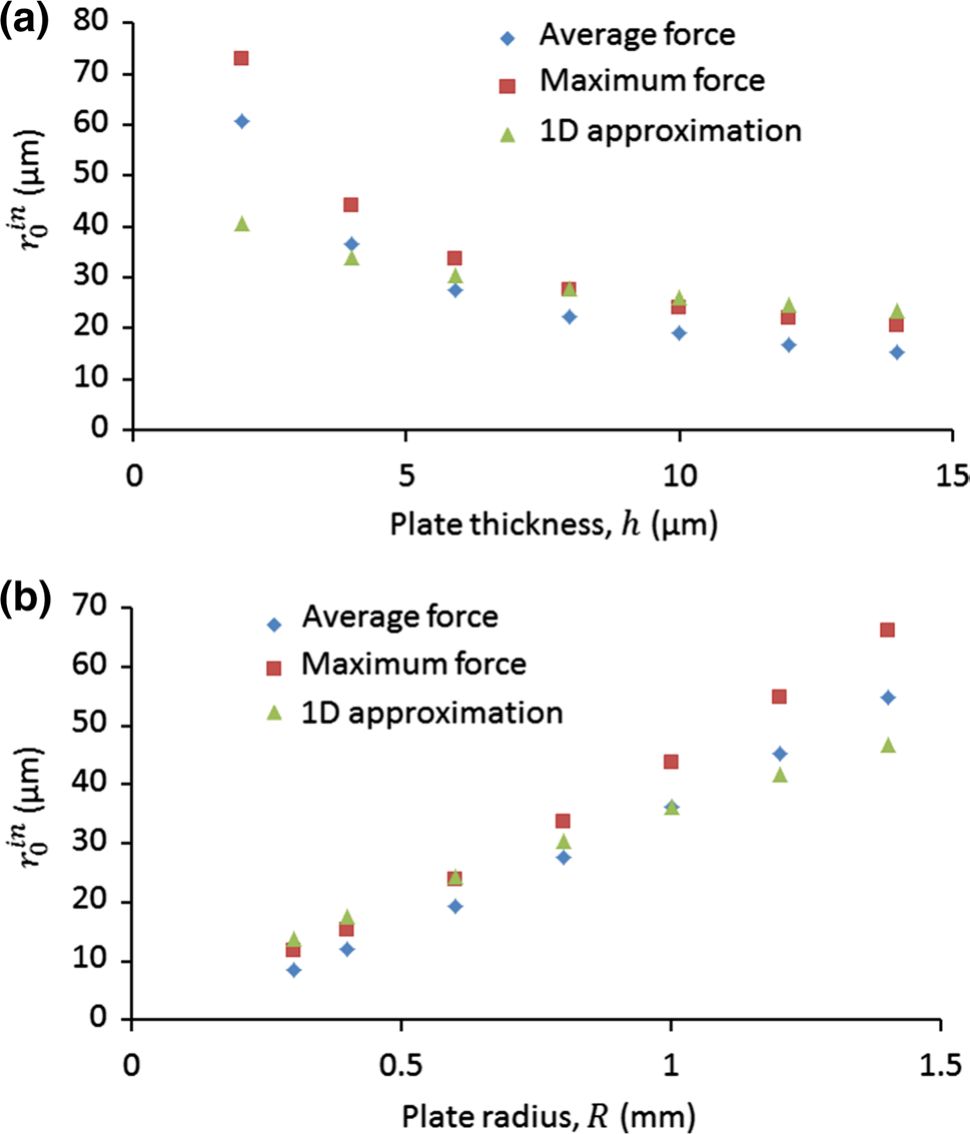
(Colour online) Comparisons on the threshold in-plane particle sizes between the modelling and theory, where the *diamonds* and *squares* show the modelled values calculated from the averaged and maximum forces over the bottom surface (with wall effect), respectively, and *triangles* show the calculated values using Eq. (27). For (**a**), the plate radius is the same, $R = 0.8$ mm, and the plate thickness is the same for (**b**), $h = 5.9$ µm

The calculated values of *r*_0_^*in*^ using Eq. (27) and those obtained from our model for various $R$ and $h$ are shown in Fig. 10. It can be seen that the modelled $r_{0}^{in}$ compare reasonably well with the calculated values under the 1D standing wave approximation. The differences between the calculated values and those modelled may be attributed to the reason that, compared to an approximated 1D standing wave, the acoustic field in the near-field is a more complex pattern. However, due to the complexity of the problem, the good comparisons between our model and the calculated values indicate that the approximated 1D standing wave may have captured the main features of (4, 1) mode and our model can be applied to study the basic physics of microparticle acoustophoresis on vibrating plate systems for even more complex vibrating modes.

## Mode switching

**Fig. 11:**
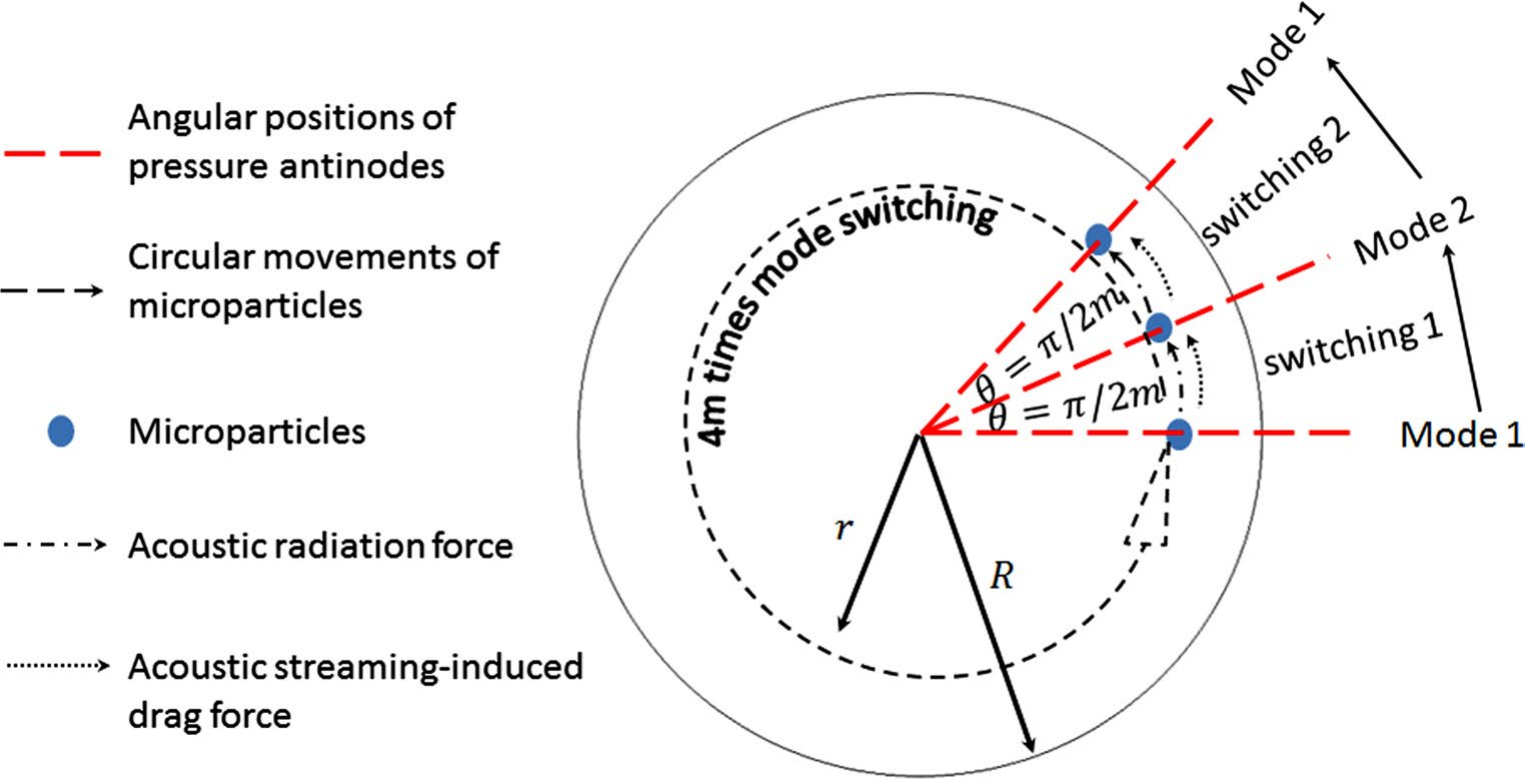
(Colour online) A schematic representation of the underlying mechanism for the circular manipulation of a single particle by continuous mode switching between two $\left( {m, n} \right)$ orthogonal modes. To complete a full circle of movement (i.e. $\theta = \pi /2m$), 4 $m$ times of mode switching are required

Eigenfrequency studies show that two orthogonal vibrating patterns for each ($m,n$) vibrating mode could be excited at two adjacent frequencies (typically hundreds of Hz difference) provided that the modes are high enough ($m \ge 1$). As shown in Fig. 11, the phase angle between two adjacent acoustic pressure antinodes of these two orthogonal patterns is

28 $$\theta = \frac{\pi }{2m}.$$

For this specific model, both the in-plane acoustic radiation force and streaming-induced drag force diverge from the vibrating nodes and converge at the vibrating antinodes, so when switching from one mode (e.g. mode 1 in Fig. 11) to the other orthogonal mode (e.g. mode 2 in Fig. 11) a particle tends to move from the vibrating antinode of the former to its closest antinode of the latter either clockwise or anticlockwise depending on the initial position of the particle (assuming the initial position of the particle slightly shifts from the vibrating antinode). The potential underlying mechanism for the circular manipulation of a single particle is schematically shown in Fig. 11. It can be seen that, for each mode switching, the particle can move by an angle, $\theta = \pi /2m$, while its distance to the centre of the circular membrane will remain the same. To complete a full circle of manipulation, 4 $m$ times of mode switching are required. This method is different from the mode switching proposed by Glynne-Jones et al. (2010) who showed that beads can be brought to any arbitrary point between the half and quarter-wave nodes when rapidly switching back and forth between half and quarter wavelength frequencies in bulk acoustofluidic devices.

## Conclusions

We have investigated the 3D acoustophoretic motion of microparticles due to acoustic radiation, acoustic streaming, gravity and buoyancy over a clamped vibrating circular plate in contact with water. The underlying physics of microparticle acoustophoresis over vibrating plates has been studied in detail. Previous predominant analyses have emphasized the in-plane acoustic streaming flows on the formation of inverse Chladni patterns, which, according to this study, may not be complete. For in-plane microparticle acoustophoresis, both the in-plane acoustic radiation forces and the in-plane streaming-induced drag forces were shown to drive microparticles to their closest vibrating antinodes. For out-of-plane microparticle acoustophoresis above vibrating antinodes, in addition to the buoyancy forces, one has to consider the acoustic radiation forces in the near-field, which prevent the out-of-plane streaming vortices from dragging microparticles away from the vibrating interface.

Based on the high efficiency of this numerical model, the threshold in-plane and out-of-plane particle sizes balanced from the acoustic radiation and streaming-induced drag force under all vibrating modes can be readily obtained. An important next step is to achieve a direct experimental verification of numerical modelling. Given a successful experimental verification, this 3D model could be extended to include the thermoviscous effects (Muller and Bruus 2014) to obtain more accurate results, but it would be very computationally expensive. According to a study by Rednikov and Sadhal (2011), the thermoviscous effects can increase the streaming velocities by 18% for water at 20 °C which, thus, will shift the threshold particle sizes.

The good comparisons between our modelling and experiments and basic theories indicate that our numerical model could be used together with high-precision experiments as a better research tool to study the many yet unsolved problems. For example, modelling suggests that mode switching between two adjacent frequencies may be used for circumferential manipulation of a single particle or a pair of particles, which might provide routes for the study of particle–particle and particle–wall interactions in acoustofluidics.

While we have shown here 3D particle size-dependent acoustophoresis over an ultrathin circular plate in water, we believe that this strategy could be applied to analyse 3D acoustophoretic motion of microparticles in other vibrating plate systems regardless of fluid medium and thickness, shape and material of plates. One particular application would be acoustophoretic handling of sub-micrometre particles, such as small cells, bacteria and viruses, whose movements are usually dominated by acoustic streaming flows. From the modelled results and the general scaling law given in Eq. (27), we can conclude that increasing plate thickness, decreasing the plate diameter and lowering the viscosity of the liquid are probably the most viable way to conduct such manipulation.

The above-mentioned applications demonstrate that our numerical model is timely and has a huge potential on studies of basic physical aspects of microparticle acoustophoresis in vibrating plate systems and the design of lab-on-a-chip devices.

## Electronic supplementary material

Below is the link to the electronic supplementary material.

Supplementary material 1 (AVI 3956 kb)

Supplementary material 2 (AVI 6685 kb)
